# Draft genome sequence of *Sphingomonas paucimobilis* strain LCT-SP1 isolated from the Shenzhou X spacecraft of China

**DOI:** 10.1186/s40793-016-0136-z

**Published:** 2016-02-24

**Authors:** Lei Pan, Hong Zhou, Jia Li, Bing Huang, Jun Guo, Xue-Lin Zhang, Long-Cheng Gao, Chou Xu, Chang-Ting Liu

**Affiliations:** Space Biomedical Laboratory, Nanlou Respiratory Diseases Department, Chinese PLA General Hospital, Beijing, 100853 China; Department of Respiratory and Critical Care Medicine, Affiliated Hospital of Binzhou Medical University, Binzhou, 256603 China; Key Laboratory of Bio-Inspired Smart Interfacial Science and Technology of Ministry of Education, Key Laboratory of Beijing Energy, School of Chemistry and Environment, Beihang University, Beijing, 100191 China

**Keywords:** genome sequence, *Sphingomonas paucimobilis*, corrosion

## Abstract

*Sphingomonas paucimobilis* strain LCT-SP1 is a glucose-nonfermenting Gram-negative, chemoheterotrophic, strictly aerobic bacterium. The major feature of strain LCT-SP1, isolated from the Chinese spacecraft Shenzhou X, together with the genome draft and annotation are described in this paper. The total size of strain LCT-SP1 is 4,302,226 bp with 3,864 protein-coding and 50 RNA genes. The information gained from its sequence is potentially relevant to the elucidation of microbially mediated corrosion of various materials.

## Introduction

*Sphingomonas paucimobilis* strain LCT-SP1 is a glucose-nonfermenting Gram-negative, chemoheterotrophic, strictly aerobic bacterium [1]. LCT-SP1, based on 16S rRNA gene sequences, is most closely related to *Sphingomonas haloaromaticamans*, which is isolated from water and soil. Several studies suggest that *S. paucimobilis* can degrade many compounds or materials, such as ferulic acid [2], lignin [3], and biphenyl [4]. LCT-SP1 was isolated from the condensate water in the Chinese spacecraft Shenzhou X.

LCT-SP1 can corrode numerous materials including epoxy resin, ester polyurethane, and ethers polyurethane. Therefore, the strain may be a suitable model for examining the properties of genes involved in microbial corrosion of materials used in aerospace applications. This study mainly aims to describe the draft genome of *S. paucimobilis* strain LCT-SP1 together with the genomic sequencing and annotation, which may be helpful in investigating the possible mechanisms in the microbial corrosion of materials.

## Organism information

### Classification and features

**Fig. 1:**
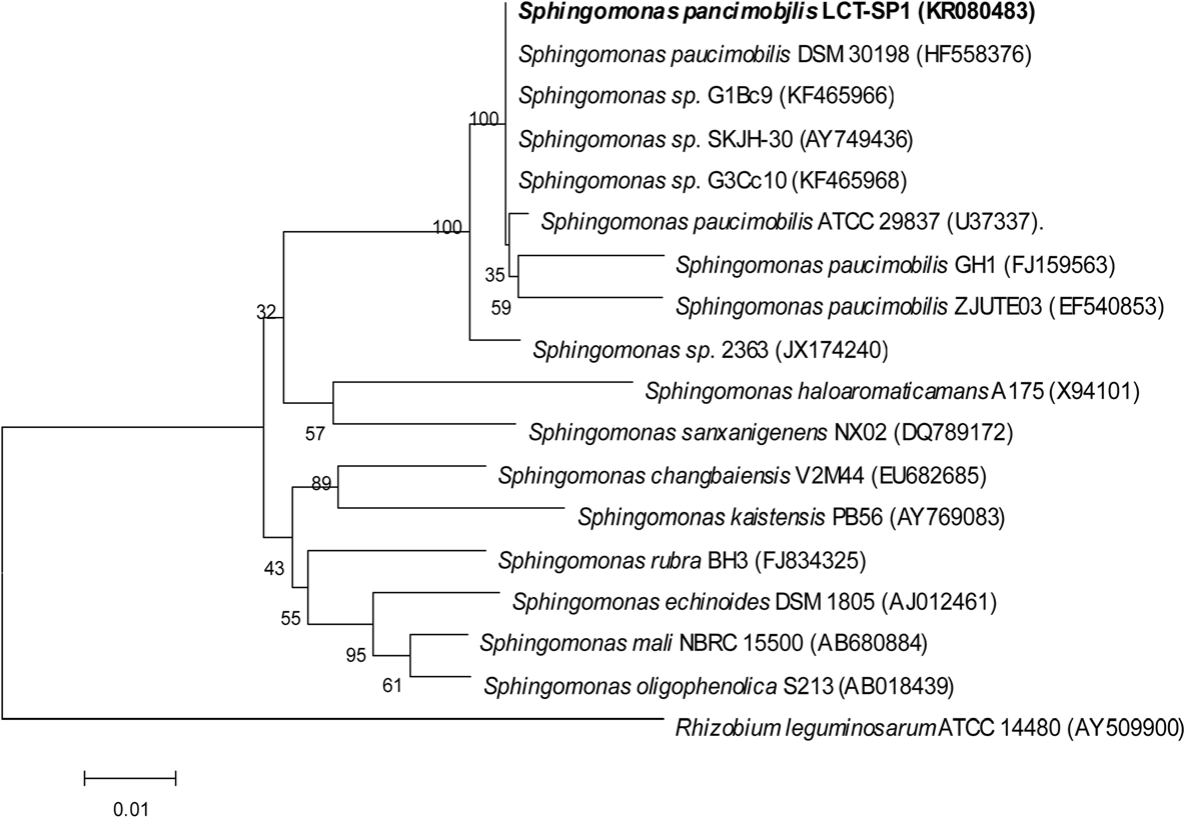
Phylogenetic tree highlighting the position of the *Sphingomonas paucimobilis* strain LCT-SP1 relative to selected *Sphingomonas* species using the *Rhizobium leguminosarum* ATCC 14480 as the outgroup. The strains and their corresponding GenBank accession numbers of 16S rRNA genes are indicated. Bar: 0.01 substitutions per nucleotide position

A phylogenetic tree was constructed with MEGA 5 [5] along with the sequences of representative members of the genus *Sphingomonas* using the maximum likelihood method based on 16S rRNA gene phylogeny (Fig. 1). Figure 1 shows that LCT-SP1 is most closely related to *Sphingomonas* sp*.*DSM 30198 (HF558376), G1Bc9 (KF465966), SKJH-30 (AY749436), and G3Cc10 (KF465968), with a sequence similarity of 100 % based on BLAST analysis. In addition, considering that the ANI is an important index in terms of phylogenetic analysis [6], the ANIs between LCT-SP1 and *Sphingomonas paucimobilis*NBRC 13935 were also calculated. The ANI result was 99.68 %, which is greater than 95 % (the species ANI cutoff value). Therefore, LCT-SP1 is asssumed to belongs to the species of *Sphingomonas paucimobilis*.

**Fig. 2:**
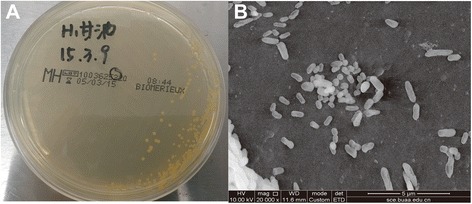
Images of the *Sphingomonas paucimobilis* strain LCT-SP1: (**a**) colonies of the strains on Luria Bertani agar plates, and (**b**) scanning electron micrographs of the strain

The general information of LCT-SP1 is shown in Table 1. LCT-SP1 is an aerobic, Gram-negative, rod-shaped, glucose-nonfermenting, slowly motile, and non-sporulating bacterium (Fig. 2-b). The strain grew optimally in the following conditions: pH 7.2, 35 °C, and at low salinity (NaCl range 0–1.0 %). On aerobic LB agar, LCT-SP1 formed several small, yellow-pigmented, round colonies (Fig. 2-a). LCT-SP1 was able to use a range of carbon substrates including D-glucose, maltose, lactose, sucrose, fucose, malic acid, acetic acid, and Tween-40.

**Table 1:** Classification and general features of *Sphingomonas paucimobilis* strain LCT-SP1 according to the MIGS recommendations [22]

MIGS ID	Property	Term	Evidence code^a^
	Classification	Domain *Bacteria*	TAS [23]
		Phylum *Proteobacteria*	TAS [24]
		Class *Alphaproteobacteria*	TAS [25, 26]
		Order *Sphingomonadales*	TAS [25, 26]
		Family *Sphingomonadaceae*	TAS [27, 28]
		Genus *Sphingomonas*	TAS [27, 28]
		Species *Sphingomonas paucimobilis*	TAS [1, 29]
		(Type) strain: LCT-SP1	IDA
	Gram stain	Negative	TAS [1]
	Cell shape	Rod-shaped	TAS [1]
	Motility	Slow motility	TAS [1]
	Sporulation	Non-sporulating	TAS [1]
	Temperature range	30-38 °C	NAS
	Optimum temperature	35 °C	NAS
	pH range; Optimum	6.0-7.5; 7.2	IDA
	Carbon source	D-glucose, maltose, lactose, sucrose, fucose, malic acid, acetic acid, Tween-40	IDA
MIGS-6	Habitat	Space cabin surface	IDA
MIGS-6.3	Salinity	0-1.0 % NaCl (w/v)	IDA
MIGS-22	Oxygen requirement	Aerobic	TAS [1]
MIGS-15	Biotic relationship	Free-living	NAS
MIGS-14	Pathogenicity	Opportunistic pathogen	TAS [1, 30, 31]
MIGS-4	Geographic location	Inner Mongolia, China	IDA
MIGS-5	Sample collection	June 5, 2013	NAS
MIGS-4.1	Latitude	Not recorded	
MIGS-4.2	Longitude	Not recorded	
MIGS-4.4	Altitude	Not recorded	

^**a**^Evidence codes -*IDA* Inferred from Direct Assay, *TAS* Traceable Author Statement (i.e., a direct report exists in the literature), *NAS* Non-traceable Author Statement (i.e., not directly observed for the living, isolated sample, but based on a generally accepted property for the species, or anecdotal evidence). These evidence codes are from the Gene Ontology project [32]

## Genome sequencing information

### Genome project history

A summary of the main project information of the *S. paucimobilis* strain LCT-SP1 is shown in Table 2. This organism was isolated from the condensate water in the Shenzhou X spacecraft, and was selected for sequencing for its phylogenetic affiliation with a lineage of *S. paucimobilis*. The genome sequences of this organism were deposited in GenBank under accession number KR080483, which belongs to the 16s ribosomal RNA coding gene sequence of LCT-SP1.

**Table 2:** Project information

MIGS ID	Property	Term
MIGS-31	Finishing quality	Improved high quality draft
MIGS-28	Libraries used	One 300bp Illumina genomic library
MIGS-29	Sequencing platforms	Illumina HiSeq2000
MIGS-31.2	Fold coverage	50×
MIGS-30	Assemblers	SOAPdenovo 1.05
MIGS-32	Gene calling method	Glimmer 3.0
	Locus Tag	ACJ66
	Genbank ID	LDUA01000000
	Genbank Date of Release	June 18, 2015
	GOLD ID	Gs0115809
	BIOPROJECT	PRJNA282437
MIGS-13	Source Material Identifier	LCT-SP1
	Project relevance	Environment

### Growth conditions and genomic DNA preparation

*S. paucimobilis* strain LCT-SP1 was grown overnight on an aerobic LB agar plate at 35 °C. The total genomic DNA was extracted from 20 mL of cells using a CTAB bacterial genomic DNA isolation method [7] with kits provided by Illumina Inc. according to the manufacturer's instructions. DNA quality and quantity was determined by spectrophotometry.

### Genome sequencing and assembly

The genome of LCT-SP1 was sequenced using paired-end sequencing technology [8] with Illumina HiSeq2000 (Illumina, SanDiego, CA, USA) at Majorbio Bio-pharm Technology Co., Ltd. (Shanghai, China). Draft assemblies were based on 6,986,766 readings, totaling 1,754 Mbp of 300 bp the PCR-free library, and 3,442,511 readings, totaling 1,556 Mbp of the 6,000 bp index library.

The assembly was performed using the SOAPdenovo software package version 1.05 [9]. The gaps among scaffolds were closed by custom primer walks or by PCR amplification, followed by DNA sequencing to achieve optimal assembly results. The genome contained 3,884 candidate protein-encoding genes (with an average size of 958 bp), giving a coding intensity of 87.7%. A total of 1,906 proteins were assigned to 25 COG families [10]. A total of 47 tRNA genes and 3 rRNA genes were identified.

### Genome annotation

Protein-coding genes of the draft genome assemblies were established using Glimmer version 3.0 [11]. The predicted CDSs were translated and employed to search the KEGG, COG, String, NR, and GO databases. These data sources were brought together to assert a product description for each predicted protein. tRNAs and rRNAs were predicted using tRNAscan-SE [12] and RNAmmer [13], respectively. Automatic gene annotation was performed by the National Center for Biotechnology Information Prokaryotic Genomes Automatic Annotation Pipeline [14].

## Genome properties

**Fig. 3:**
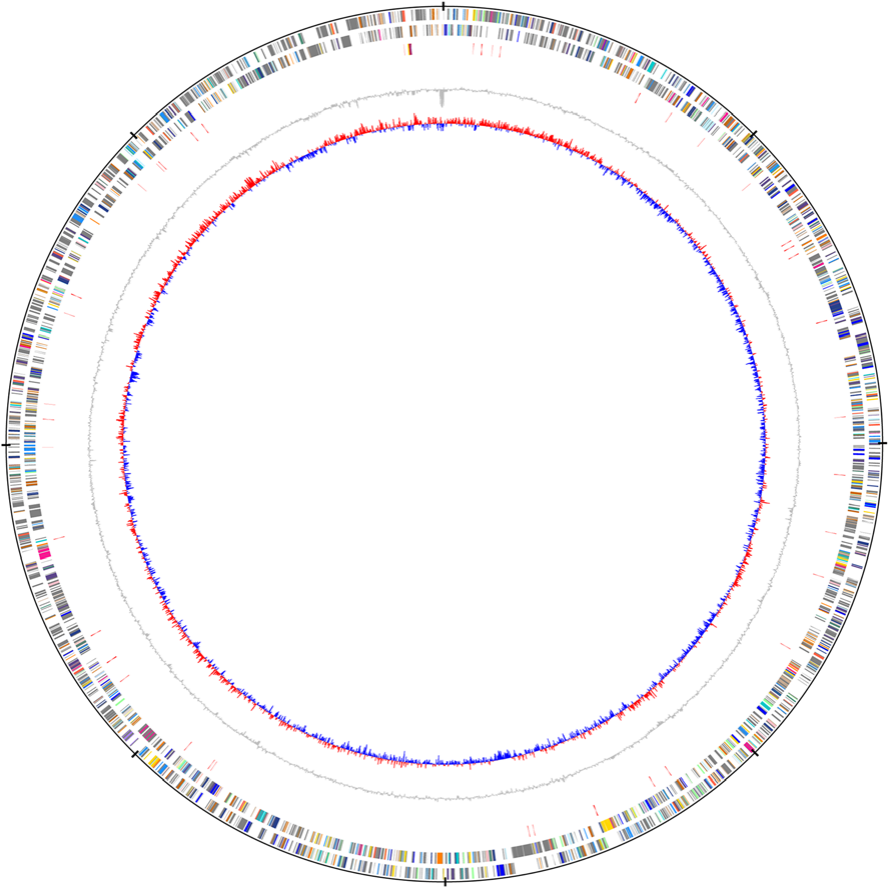
Circular map of the draft genome of the *Sphingomonas paucimobilis* strain LCT-SP1. From outside to the center: Genes on the forward strand (colored by the predicted coding sequences), genes on the reverse strand (colored by COG categories), RNA genes, GC content, and GC skew. The map was created using the DNAPlotter according to the method described by Carver *et al*. (2009) [33]. DNAPlotter reads the common sequence formats (EMBL, Genbank, GFF) using the Artemis file-reading library and displays the sequence as the circular plot. Additional feature files can be read in and overlaid on the sequence

The LCT-SP1 genome consisted of 4,302,226 bp circular chromosomes with a GC content of 65.66 % (Table 3). Of the 3,934 predicted genes, 3,884 (98.73 %) were protein-coding genes, and 50 (1.27 %) were RNA genes (3 rRNA genes, and 47 tRNA genes). In addition, among the total predicted genes, 1,906 (48.45 %) represented COG functional categories. Of these, the most abundant COG category was “General function prediction only” (211 proteins) followed by “Amino acid transport and metabolism” (171 proteins), “Translation” (141 proteins), “Energy production and conversion” (140 proteins), “Replication, recombination and repair” (130 proteins), “Function unknown” (124 proteins), “Inorganic ion transport and metabolism” (210 proteins), and “Replication, recombination and repair” (201 proteins). The properties and statistics of the genome are summarized in Table 3. The draft genome map of *S. paucimobilis* strain LCT-SP1 is illustrated in Fig. 3, and the distribution of genes into COG functional categories is presented in Table 4.

**Table 3:** Genome statistics

Attribute	Value	% of total
Genome size (bp)	4,302,226	100.00
DNA coding (bp)	3,772,440	87.69
DNA G + C (bp)	2,824,842	65.66
DNA scaffolds	91	100.00
Total genes	3,934	100.00
Protein coding genes	3,884	98.73
RNA genes	50	1.27
Pseudo genes	0	0.00
Genes in internal clusters	1,610	40.93
Genes with function prediction	3,911	99.42
Genes assigned to COGs	1,906	48.45
Genes with Pfam domains	2,571	65.35
Genes with signal peptides	367	9.33
Genes with transmembrane helices	846	21.50
CRISPR repeats	6	-

**Table 4:** Number of genes associated with general COG functional categories

Code	Value	% age	Description
J	141	3.58	Translation, ribosomal structure and biogenesis
A	0	0.00	RNA processing and modification
K	115	2.92	Transcription
L	130	3.30	Replication, recombination and repair
B	1	0.03	Chromatin structure and dynamics
D	14	0.36	Cell cycle control, Cell division, chromosome partitioning
V	27	0.69	Defense mechanisms
T	78	1.98	Signal transduction mechanisms
M	75	1.91	Cell wall/membrane biogenesis
N	27	0.69	Cell motility
U	57	1.45	Intracellular trafficking and secretion
O	89	2.26	Posttranslational modification, protein turnover, chaperones
C	140	3.56	Energy production and conversion
G	109	2.77	Carbohydrate transport and metabolism
E	171	4.35	Amino acid transport and metabolism
F	47	1.19	Nucleotide transport and metabolism
H	94	2.39	Coenzyme transport and metabolism
I	85	2.16	Lipid transport and metabolism
P	114	2.90	Inorganic ion transport and metabolism
Q	57	1.45	Secondary metabolites biosynthesis, transport and catabolism
R	211	5.36	General function prediction only
S	124	3.15	Function unknown
-	2,028	51.55	Not in COGs

## Insights from the genome sequence

Several studies suggest that the genus *S. paucimobilis* can degrade many compounds or materials, such as ferulic acid [2], lignin [3], and biphenyl [4]. Arens *et al.* believed that the localized corrosion of copper cold-water pipes resulted from the genus *Sphingomonas*, leading to surface erosions, covered tubercles, and through-wall pinhole pits on the inner surface of the pipe [15]. *S. paucimobilis* strain LCT-SP1 can corrode several materials including epoxy resin, ester polyurethane, and ethers polyurethane (unpublished data). LCT-SP1 was isolated from the condensation water in the Chinese spacecraft Shenzhou X. Therefore, LCT-SP1 could be a suitable model for studying the properties of genes involved in microbial corrosion of aerospace related materials.

Additionally, EC 1.14.11.2, *gloA*, and *arsC* gene were present in LCT-SP1, which was identified with 100% similarity to *Sphingomonas* sp. S17 [16]. EC 1.14.11.2 is categorized as a procollagen-proline catalyzing enzyme [17]. The *gloA* gene encodes a glyoxalase that can reduce methylglyoxal toxicity in a cell [18]. Furthermore, *arsC* gene produces an arsenate reductase that can convert arsenate into arsenite, which is accordingly exported from cells by an energy-dependent efflux process [19]. Therefore, the genes mentioned above are likely responsible for the ability of LCT-SP1 to degrade various recalcitrant aromatic compounds and polysaccharides.

The LCT-SP1 genome also contained an *NhaA*-type CDS for the Na^+^/H^+^ antiporter and some subunits of the multisubunit cation antiporter (Na^+^/H^+^) [20], which suggested that this strain should be compatible with its alkaline and hypersaline environment, and could corrode metallic materials by changing the pH balance of their surface.

Also, biofilms from bacteria may be beneficial for corrosion control because of the removal of corrosive agents and the generation of a protective layer by biofilms [21]. LCT-SP1 included the gene encoding biofilm dispersion protein BdlA and biofilm growth-associated repressor that could inhibit the formation of biofilm, which may explain the microbial corrosion of materials. Further studies are needed to investigate these corrosion-based gene-coding sequences to reveal the role of LCT-SP1 in the microbial corrosion of materials.

## Conclusions

The genome of *S. paucimobilis* strain LCT-SP1 isolated from the condensate water in the Chinese spacecraft Shenzhou X was sequenced. The strain LCT-SP1 genome included numerous genes that are likely responsible for their ability to degrade various recalcitrant aromatic compounds and polysaccharides. Further study of these corrosion-based gene-coding sequences may reveal the role of *S. paucimobilis* LCT-SP1 in microbial corrosion of materials, especially in aerospace applications. The genome sequence has been deposited at DDBJ/EMBL/GenBank under accession number LDUA00000000.

## Abbreviations

ANI: Average nucleotide identity
LB: Luria–Bertani
CTAB: Cetyl Trimethyl Ammonium Bromide
COG: Clusters of orthologous group
CDS: Coding sequences
